# TRIM23 overexpression is a poor prognostic factor and contributes to carcinogenesis in colorectal cancer

**DOI:** 10.1111/jcmm.15203

**Published:** 2020-03-30

**Authors:** Yudong Han, Ye Tan, Yuanyuan Zhao, Yongchun Zhang, Xinjia He, Li Yu, Haiping Jiang, Haijun Lu, Haiying Tian

**Affiliations:** ^1^ Department of Thoracic Surgery Affiliated Hospital of Qingdao University Qingdao China; ^2^ Department of Radiation Oncology Affiliated Hospital of Qingdao University Qingdao China

**Keywords:** cell cycle, colorectal cancer, P53, proliferation, TRIM23

## Abstract

The tripartite motif (TRIM) family proteins play a great role in carcinogenesis. However, the expression pattern, prognostic value and biological functions of tripartite motif containing 23 (TRIM23) in colorectal cancer (CRC) are poorly understood. Here, we found that TRIM23 is up‐regulated and associated with tumour size, lymph node metastasis, American Joint Committee on Cancer (AJCC) stage and poor prognosis in CRC. Multivariate Cox regression analyses revealed that TRIM23 overexpression could be identified as an independent prognostic factor for CRC. TRIM23 could promote the proliferation of CRC cell in vitro and in vivo; additionally, TRIM23 depletion induced G1­phase arrest. Gene set enrichment analysis (GSEA) revealed that P53 and cell cycle signalling pathway‐related genes were enriched in patients with high TRIM23 expression levels. We show in this study that TRIM23 physically binds to P53 and enhances the ubiquitination of P53, thereby promoting tumour proliferation. Thus, our data indicated that TRIM23 acts as an oncogene in colorectal carcinogenesis and may provide a novel therapeutic target for CRC management.

## 1 INTRODUCTION

Colorectal cancer ranks third in morbidity and in mortality of all human cancers worldwide.^1^ Patients with advanced colorectal cancer have a poor prognosis and eventually die after operation as a result of recurrence and metastasis. As characterized, unrestrained and rapid cell proliferation is a significant characteristic of malignant tumours, not only for the growth and development of primary tumour, but also for metastatic tumour cells in target organs.^2^, ^3^ Pathologic classification is currently used to assess prognosis and inform the treatment of colorectal cancer.^4^ Identification of the molecular mechanism of tumorigenesis and metastasis will advance the development of anti‐colorectal cancer strategies. Therefore, more research is needed to develop more effective biomarkers for diagnosis and treatment of CRC.

The tripartite motif family proteins are evolutionarily conserved proteins, consist of one or two B‐box domains, a RING finger domain and a coiled‐coil domain.^5^, ^6^ Tripartite motif family proteins have been proposed to be involved in various biological processes,^7^, ^8^ and their alterations often lead to diverse clinical disorders, including various human cancers.^9^ Emerging clinical evidence reveals that the disorder of ubiquitin‐mediated degradation of tumour suppressors is likely to be involved in carcinogenesis.^10^ Because contains a RING finger domain, most TRIM proteins could be defined as E3 ubiquitin ligases which have been viewed as the potential therapeutic target in various cancer.^11^ For instance, TRIM proteins including TRIM25,^12^ TRIM37,^13^ TRIM27,^14^ TRIM47,^15^ TRIM59^16^ and TRIM65^17^ were shown to be involved in prostate, breast, colorectal, gastric and bladder urothelial cancer through the ubiquitin‐mediated degradation, indicating vital function of the TRIM proteins in carcinogenesis. As a member of the TRIM family, TRIM23 participates in many pathophysiological processes.^18^, ^19^ Higher TRIM23 expression was reported to be associated with hepatocellular carcinoma^20^ and gastric cancer.^21^ However, the expression pattern and biological functions of TRIM23 remains poorly understood in CRC.

In the current research, we explored the biological functions, clinical application and potential molecular mechanisms of TRIM23 in CRC. TRIM23 expression was significantly increased in colorectal cancer tissues compared with normal adjacent tissues. Overexpression of TRIM23 in patients with CRC was accompanied by poor postoperative survival. Gene set enrichment analysis (GSEA) confirmed that TRIM23 acts as an oncogene to regulate cell proliferation through p53‐cell cycle signalling pathway, which was further identified in colorectal cancer cells. Collectively, our study has identified that TRIM23 is a potent prognostic factor and a potential target for the treatment of CRC.

## 2 MATERIALS AND METHODS

### 2.1 Patients and tissue samples

Tumour tissues with clinicopathological information and the normal adjacent tissues were obtained from patients with colorectal cancer who underwent surgery at Affiliated Hospital of Qingdao University from January 2003 to January 2007. Sixty fresh samples of CRC and paired normal adjacent tissues were frozen in liquid nitrogen within 10 minutes and stored at −80°C until quantitative real‐time polymerase chain reaction was performed. Informed consent was obtained from patients and their families. This study was approved by the ethics committee of Affiliated Hospital of Qingdao University.

### 2.2 Cell culture and treatment

Human CRC cell lines (SW480, HT29, SW1116, HCT116, SW620, FHC) were purchased from the Cell Bank of Chinese Academy of Science. According to ATCC protocols, cells were cultured in Roswell Park Memorial Institute‐1640 (Life Technologies) at 37°C in a humidified incubator under 5% CO2 conditions. Foetal bovine serum (10%) (FBS, Life Technologies) and 1% penicillin/streptomycin (Life Technologies) were supplemented into the culture media.

Colorectal cancer cells were transfected with two small interfering RNA (siRNA) targeting TRIM23 and a non‐specific control siRNA sequence (siNC) using Lipofectamine 2000 reagent (Invitrogen) according to the manufacture's instruction. The siRNAs were synthesized by Genepharm Technologies. The TRIM23‐overexpressing plasmid and the empty plasmid which purchased from GENEray Biotechnology were transfected into cells using the FuGENE Transfection Reagent (Promega, E2311).

### 2.3 Immunohistochemistry

To detect the TRIM23 expression level in CRC tissues and normal adjacent tissues, streptavidin peroxidase immunohistochemistry was used. All procedures were executed in accordance with the manufacturer's instructions. The sections were incubated at 4°C overnight with rabbit anti‐human TRIM23 antibody (1:100; HPA039605, Sigma) after deparaffinization and antigen retrieval. Then, the sections were incubated with a drop of biotin‐labelled secondary antibody for 30 minutes at 37°C. We subsequently added a drop of horseradish peroxidase‐conjugated streptomycin working solution to the sections and incubated them for 30 minutes at 37°C. Visualization was performed with 3,3′‐diaminobenzidine (DAB), and sections were counterstained with haematoxylin.^22^

TRIM23 expression was assessed with regard to the percentage of positive cells and the staining intensity. The staining intensity was scored as follows: 0 for no intensity, 1 for no or very weak staining, 2 for moderate intensity and 3 for strong intensity. The percentage of positive cells was rated as follows: 0 for <10%, 1 for 10% to <25%, 2 for 25% to <50% and 3 for >50%. Total histological score = staining intensity + percentage of positive cells. The patients could be classified into two groups: low‐expression group (0‐2) and high‐expression group (3‐6).

### 2.4 Quantitative real‐time PCR

Total RNA of specimens and cells harvested and washed with PBS was extracted with TRIzol reagent (Invitrogen). Samples with 260/280 values of 1.8‐2.0 were considered for further research. cDNA was then reverse transcribed with oligodT using M‐MLV reverse transcriptase (Thermo Fisher Scientific). Quantitative real‐time PCR was performed with Maxima SYBR Green qPCR Master Mixes (Thermo Fisher Scientific) in an ABI 7300 system (Applied Biosystem). The relative expression levels of genes of interest were determined using the 2−ΔΔCt method, and GAPDH served as an internal control. The primers used are listed in Table S1.

### 2.5 Western blot analysis

RIPA lysis buffer (Thermo Fisher) with protease inhibitor (Sigma) was used to extract the total protein from cancer cells. The same amounts of total proteins were separated via 10% sodium dodecyl sulphate‐polyacrylamide (SDS) gel electrophoresis and then transferred to polyvinylidene fluoride (PVDF, Millipore) membranes. After the membranes were blocked with 5% skim milk, the membranes were incubated with the primary antibodies overnight at 4°C and then with the horseradish peroxidase‐conjugated secondary antibody (Santa Cruz Biotechnology). Signals were detected with enhanced chemiluminescence system (Bio‐Rad). Primary antibodies were obtained from the following companies: TRIM23 from Sigma; P53, P21, CDK4, CDK6, cyclin D1 and GAPDH from Cell Signaling Technology.

### 2.6 Cell viability assays

Control and treated cells were seeded in 96‐well plates at a density of 1 × 10^4^ cells/well and then incubated for 24, 48 or 72 hours. Following the manufacturer's instructions, cell viability was assessed using a Cell Counting Kit‐8 (Dojindo Lab). A microplate reader (Bio‐Rad) was used to measure the optical density values of each well at a wavelength of 450 nm.

### 2.7 Cell cycle distribution assay

Propidium iodide (PI, Sigma) staining was used to analyse the cell cycle. Treated and untreated cells were harvested by trypsinization, washed in PBS and fixed with ice‐cold 70% ethanol at −20°C for at least 2 hours. After washing with PBS, cells were incubated with ribonuclease (Sigma) at 37°C for 15 minutes and then incubated with PI (0.05 mg/mL, Sigma) in the dark for 30 minutes at room temperature. Finally, a flow cytometer (BD Biosciences) was used to analyse DNA content.

### 2.8 In vivo xenograft assay

The experimental procedures were approved by the Institutional Animal Care and Use Committee of Affiliated Hospital of Qingdao University. Male BALB/c‐nu mice (4‐5 weeks old, 18‐20 g) were purchased from Shanghai Laboratory Animal Company (SLAC). For each cell line, cell suspensions (2 × 10^6^ cells) in a total volume of 100 μL were injected subcutaneously into the right and left flank of nude mice. Tumour length and width were measured and recorded every 4 days starting 2 weeks after inoculation. Tumour volume was calculated as 1/2 × length × width^2^.

### 2.9 Immunofluorescence, Co‐immunoprecipitation and ubiquitination assay

To prepare slides of colorectal cancer cell lines for immunofluorescent staining, cells were plated on cover slides in 24‐well plates and allowed to grow for 24 hours. The slides were then fixed in 4% formalin for 15 minutes at 4°C and rinsed by PBS three times. The cover slides were first treated with 0.5％ Triton for 10 minutes and blocked with 10% goat serum at room temperature for 1 hour. Primary antibody incubations were performed overnight at 4°C. After extensive washing with PBS, secondary antibody was applied to the sections at room temperature for 1 hour. Slides were washed with PBS three times and then mounted with VECTASHIELD mounting medium (Vector laboratories, Inc H‐1200).

Co‐immunoprecipitation (COIP) was performed using a Pierce™ Co‐Immunoprecipitation Kit (Thermo Scientific), following the manufacturer's instructions. Both the input and IP samples were analysed by Western blotting.

### 2.10 Gene set enrichment analysis (GSEA)

Gene set enrichment analysis is a method of analysing and interpreting microarray and similar data using biological knowledge.^23^ In our study, the TCGA data set of CRC was analysed by GSEA2‐2.2.2 as previously described. The gene sets showing a false discovery rate (FDR) of 0.25, a well‐established cut‐off for the identification of biologically relevant gene, were considered enriched between the classes under comparison. The ‘c2.all.v5.0.symbols.gmt’ from the Molecular Signatures Database‐MSigDB was used to run GSEA, and 1000 permutations were used to calculate the *P*‐value; the ‘permutation type’ was set to ‘gene set’. All other parameters were set to default, except that the gene set should represent at least 15 genes.

### 2.11 Data analysis

The statistical analyses were performed using SPSS version 16 software, and graphical representations were generated with GraphPad Prism 5 software. The data are presented as the means ± SD, and Student's *t* test was used to evaluate differences between groups. The relationship between the clinicopathological features of tumours and the expression of TRIM23 protein was evaluated by chi‐square tests. Overall survival analyses were assessed by the Kaplan‐Meier method with the log‐rank test and Cox regression analysis. *P* < .05 was regarded as significant.

## 3 RESULTS

### 3.1 Up‐regulated TRIM23 expression associated with poor survival in CRC

**FIGURE 1:**
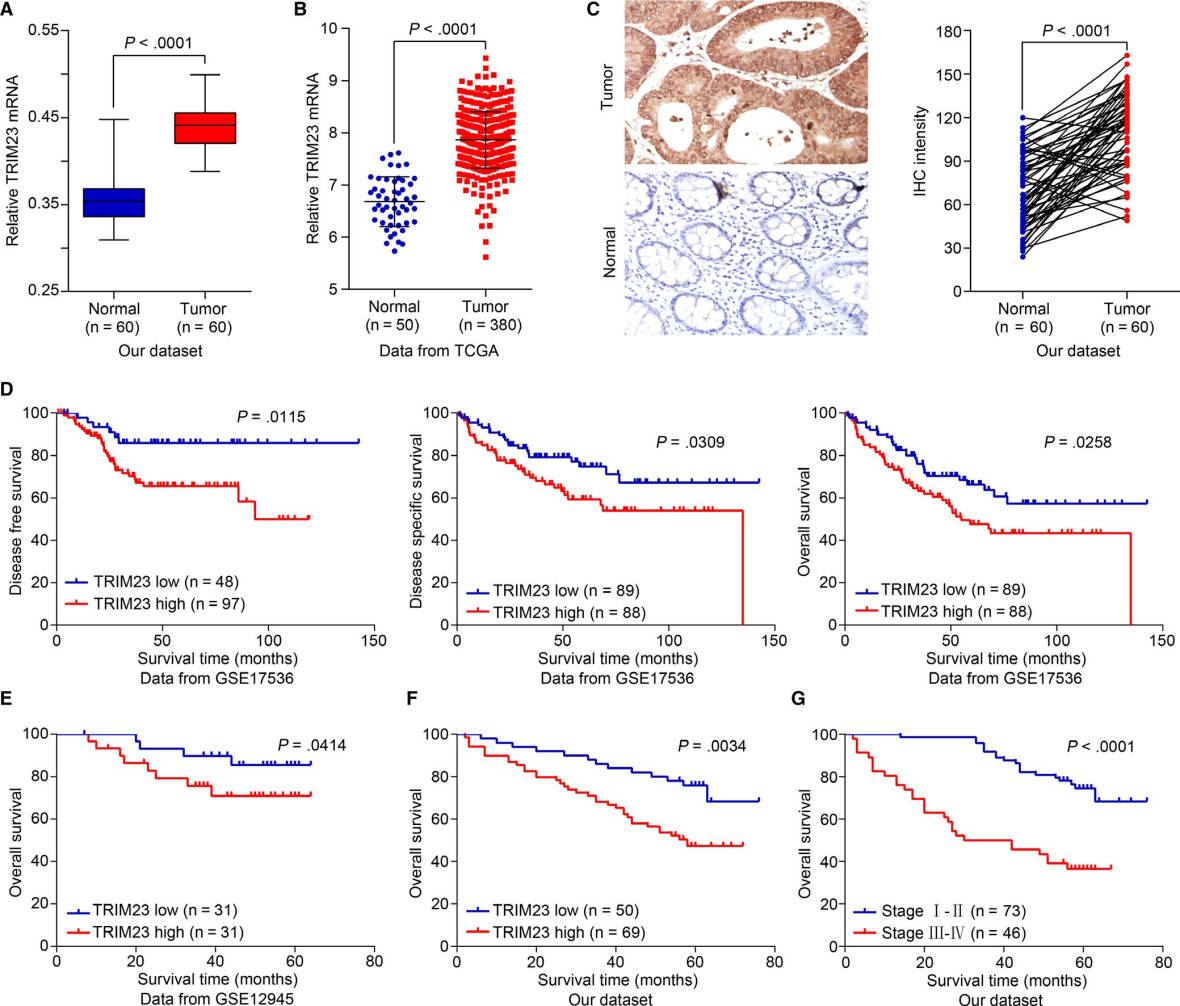
TRIM23 overexpression was associated with poor prognosis in CRC. A, The expression level of TRIM23 mRNA was higher in tumour tissues compared with normal adjacent tissues (***P < .0001). B, TRIM23 expression was significantly increased in CRC tumour tissues when compared with normal tissues from TCGA data set (***P < .0001). C, Immunohistochemical staining of TRIM23 in tumour tissues and normal adjacent tissues. D, Survival analysis of CRC patients from GEO data set (GSE17536). E, Kaplan‐Meier survival analysis of CRC patients from GEO data set (GSE12945). F, Kaplan‐Meier survival analysis of CRC patients from our hospital (*P* = .0034). G, Kaplan‐Meier survival analysis of CRC patients with different AJCC stage

To investigate TRIM23 expression patterns in CRC, we examined mRNA level of TRIM23 in 60 CRC tissues and normal adjacent tissues by real‐time PCR. Our data showed that TRIM23 mRNA levels were notably increased in tumour tissues compared with that in normal tissues (Figure 1A). Additionally, multiple microarray data sets of colorectal cancer from TCGA were analysed, and we found that the TRIM23 expression level was significantly higher in tumour tissues than in normal adjacent tissues (Figure 1B). Meanwhile, the expression of TRIM23 protein was measured by immunohistochemical staining assay in 60 CRC tissues and paired normal adjacent tissues. Based on the results, the expression of TRIM23 protein was significantly less prevalent in the normal tissues than in the CRC tissues (Figure 1C). The GEO data set (GSE17536, Figure 1D) illustrated that higher TRIM23 expression predicted poor disease‐free survival, disease‐specific survival and overall survival. Another GEO data set (GSE12945, Figure 1E) also demonstrated that patients with higher TRIM23 expression suffered poor postoperative overall survival.

Based on immunohistochemical results, we classified 119 HCC patients into two groups and found that high TRIM23 expression was dramatically associated with shortened CRC patients’ overall survival (*P* = .0034, Figure 1F). Both the widely used AJCC staging (*P* < .0001, Figure 1G) and the stratification by TRIM23 level displayed high prognostic significance. In order to verify the clinical significance of TRIM23, Table 1 summarizes the relationship between TRIM23 expression and clinicopathological features of colorectal cancer patients. TRIM23 expression was notably associated with tumour size (*P* = .031), lymph node metastasis (*P* = .016) and AJCC stage (*P* = .027). Multivariate Cox regression analyses revealed that along with AJCC stage, overexpression of TRIM23 (*P* = .027) could be considered an independent prognostic factor for CRC patients (Table 2). Collectively, these data demonstrate that TRIM23 is critical during carcinogenesis in CRC.

**TABLE 1:** Correlations between TRIM23 protein expression and clinicopathological parameters in CRC patients

Variables	No. of patients	TRIM23 expression		*P*
		High (69)	Low (50)	
Age	119	68.00 ± 10.87	64.72 ± 11.45	.115
Gender				
Male	63	35	28	.569
Female	56	34	22	
Location				
Proximal colon	61	31	30	.104
Distal colon	58	38	20	
Tumour size (cm^3^)				
<30	60	29	31	.031*
≥30	59	40	19	
T stage				
T1+T2	17	11	6	.544
T3+T4	102	58	44	
Lymph node metastasis				
Absent	68	33	35	.016*
Present	51	36	15	
Distant metastasis				
M0	115	66	49	.638
M1a	4	3	1	
Histological grade				
Ⅰ, Ⅰ‐Ⅱ, Ⅱ	86	48	38	.439
Ⅱ‐Ⅲ, Ⅲ	33	21	12	
AJCC stage				
Ⅰ‐Ⅱ	62	30	32	.027*
Ⅲ‐Ⅳ	57	39	18	

Note

*P* values are from chi‐square test and were significant at <.05.

* *P* < .05.

**TABLE 2:** Univariate and multivariable Cox regression analyses of the association between clinicopathological parameters and overall survival

Characteristics	HR (95% CI)	*P*
Univariate analysis		
Age	1.001 (0.977‐1.026)	.928
Gender	1.295 (0.730‐2.299)	.377
Tumour size (cm^3^)	1.407 (0.795‐2.489)	.241
T stage	2.095 (0.752‐5.834)	.157
Lymph node metastasis	2.503 (1.408‐4.450)	.002**
Distant metastasis	5.137 (1.812‐14.569)	.002**
Histological grade	0.883 (0.474‐1.645)	.695
TRIM23 expression	2.514 (1.324‐4.773)	.005**
AJCC stage	4.086 (2.154‐7.751)	<.001***
Multivariate analysis		
TRIM23 expression	2.158 (1.094‐4.257)	.027*
AJCC stage	3.338 (1.531‐7.278)	.002**

Note

*P* values are from chi‐square test and were significant at <.05.

* *P* < .05.

** *P* < .01.

*** *P* < .001.

### 3.2 TRIM23 promoted the proliferation of CRC cell in vitro and in vivo

**FIGURE 2:**
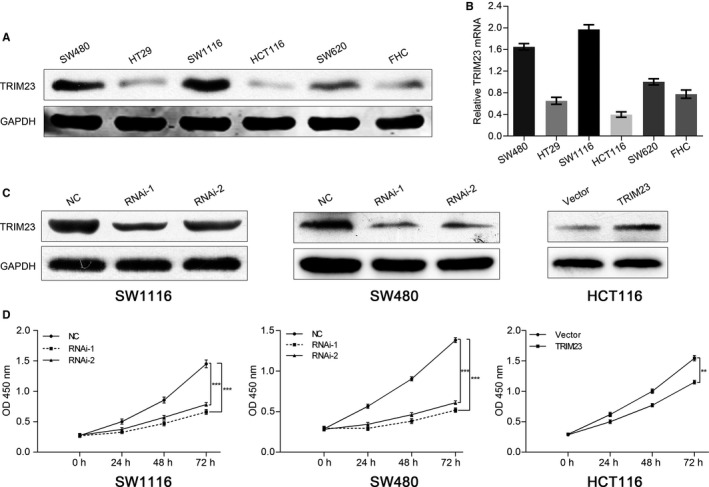
TRIM23 promotes proliferation of colorectal cancer cells in vitro. A, Western blot was performed in 6 different colorectal cancer cells (n = 3). B, Real‐time PCR was performed in 6 different colorectal cancer cells (n = 3). C, TRIM23‐siRNAs and TRIM23 overexpression plasmids were transfected into cancer cells successfully. D, Cell proliferation assay was performed in SW1116, SW480 and HCT116 cells (n = 3). NC: scrambled siRNA‐transfected cells; RNAi‐1, RNAi‐2: TRIM23‐siRNA‐transfected cells

We estimated the expression of TRIM23 in 6 different CRC cells by real‐time PCR and Western blot. The results showed SW480 and SW1116 expressed higher levels of TRIM23, while HCT116 expressed lower level of TRIM23 (Figure 2A,B). Therefore, we transfected SW480 and SW1116 with TRIM23 siRNAs and HCT116 with TRIM23‐overexpressing plasmid, respectively (Figure 2C). CCK‐8 assay showed that knock‐down of TRIM23 significantly suppressed cell proliferation in both SW480 and SW1116 cells, while TRIM23 overexpression increased cell proliferation capacity of HCT116 cells (Figure 2D).

**FIGURE 3:**
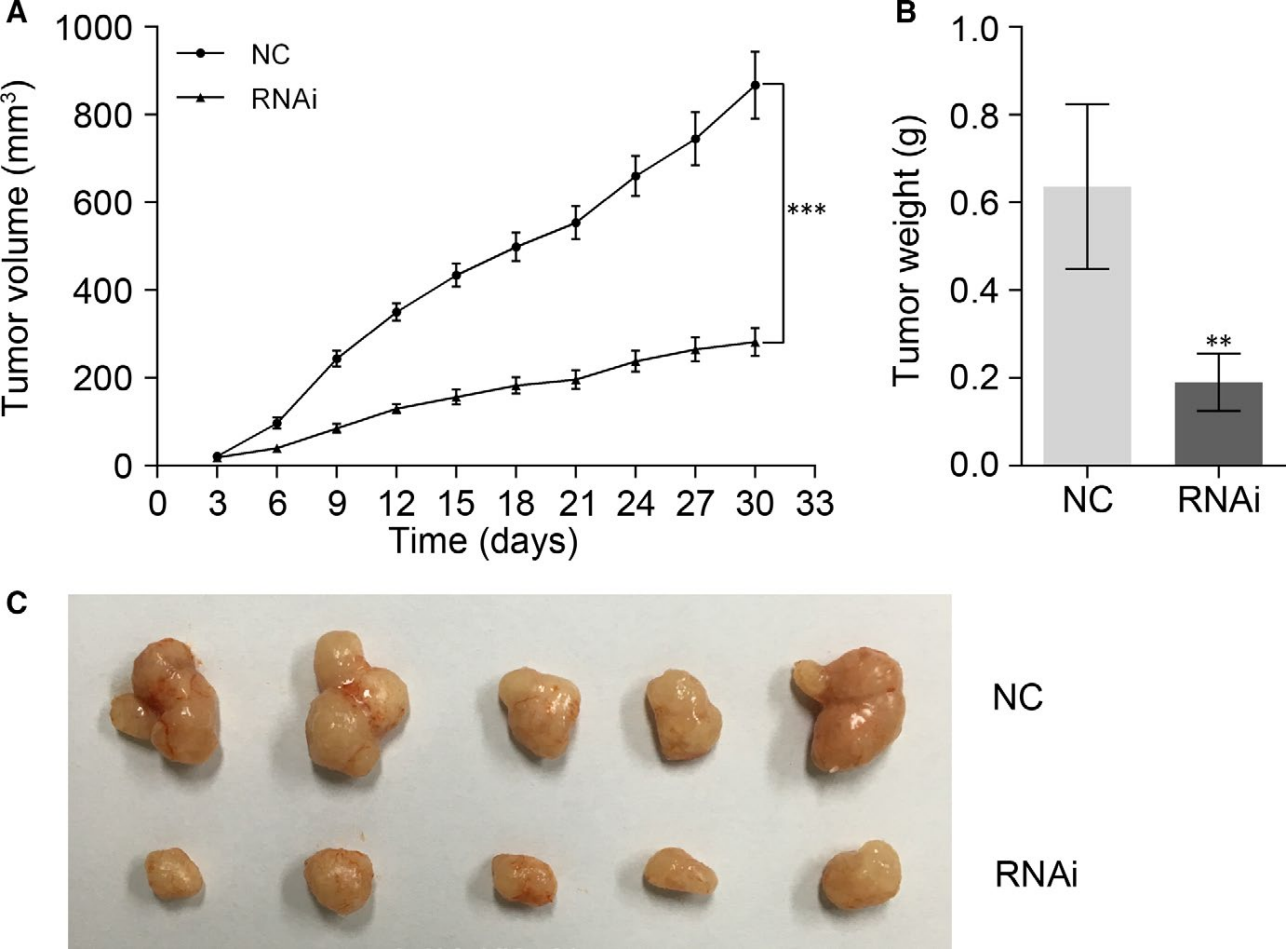
TRIM23 promotes proliferation of colorectal cancer cells in vivo. A, Tumour volume was measured after different treatments in nude mice (n = 5, ***P < .001). B and C, Mice were killed, and tumours were weighed at day 52. The five tumour tissues of the RNAi group were smaller than those from the NC group (**P < .01)

To investigate the role of TRIM23 on carcinogenicity in vivo, xenograft tumour assays were performed using SW480 cells transfected with scramble siRNA or TRIM23‐siRNA. We found that TRIM23 knock‐down obviously inhibited tumour growth rate in mice (*P* < .01, Figure 3). The weight of TRIM23‐knock‐down tumours was less than that of control tumours. Collectively, our in vitro and in vivo observed results suggest that TRIM23 functions as an oncogene in CRC.

### 3.3 Depletion of TRIM23 induced G1‐phase arrest

**FIGURE 4:**
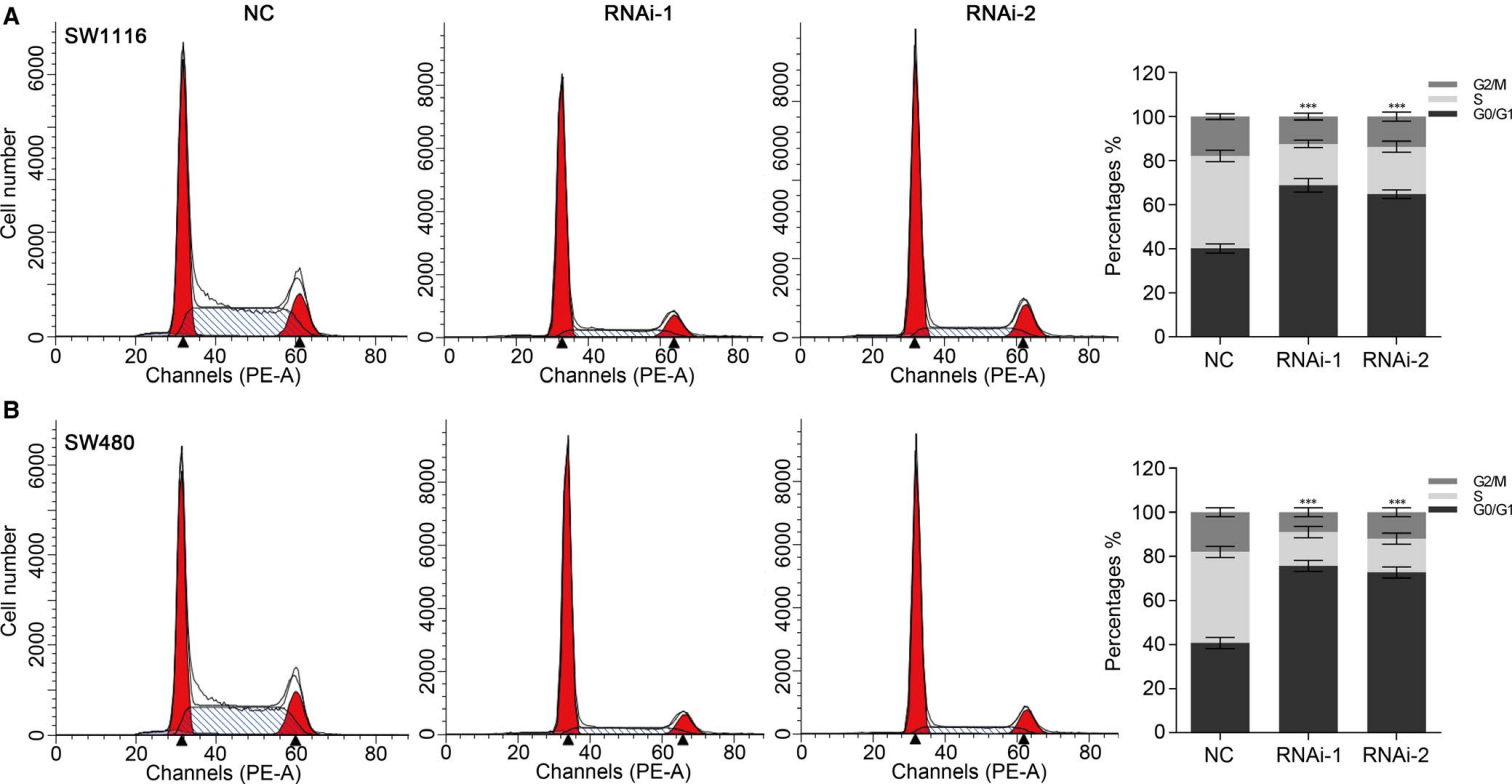
Silencing of TRIM23 induced G0/G1 arrest in CRC cells. Cell cycle profile was analysed using flow cytometry. Data were based on at least three independent experiments and are shown as the mean ± SD (***P < .001)

PI staining and flow cytometry analysis were used to assess the effects of TRIM23 knock‐down on cell cycle of CRC cells. TRIM23‐siRNA infection caused a obvious increase of G0/G1‐phase cells and a decrease of S and G2/M‐phase cells compared with the non‐specific scramble siRNA group (Figure 4A,B). The data strongly suggested that TRIM23 may promote colorectal cancer progression by regulating colorectal cancer cell proliferation.

### 3.4 Identification of potential associated signalling pathways and processes

**FIGURE 5:**
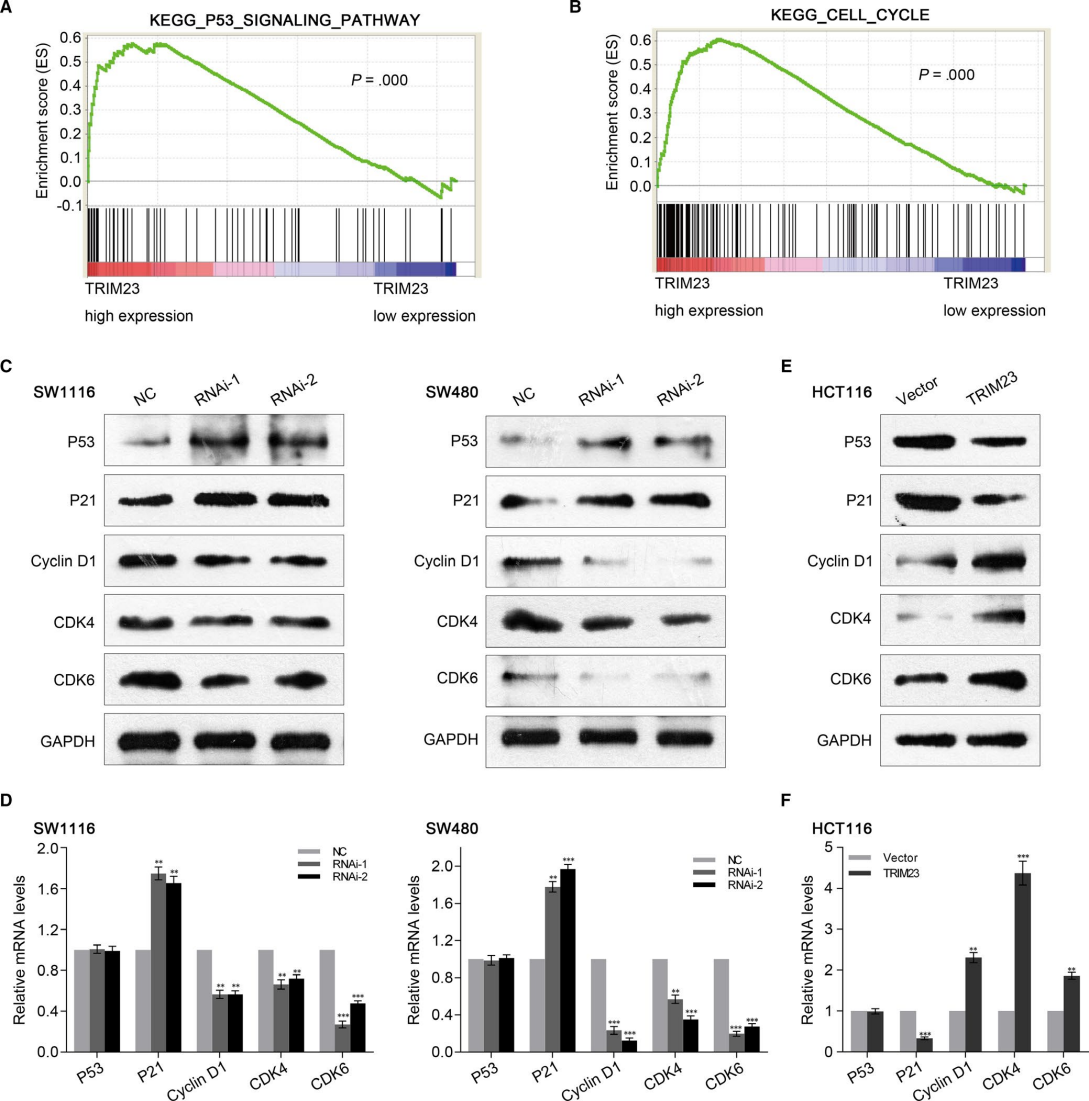
TRIM23 promotes proliferation of CRC cells through P53‐cell cycle signalling pathway. A and B, Performance of GSEA based on TCGA data set. High expression of TRIM23 correlated with P53‐cell cycle pathway. C and D, Expression of P53‐cell cycle pathway‐related gene was evaluated by Western blot and real‐time PCR in SW1116 and SW480 cells transfected with control siRNA and TRIM23 siRNAs (n = 3, **P < .01, ***P < .001). E and F, Expression of P53‐cell cycle pathway‐related gene was evaluated by Western blot and real‐time PCR in HCT116 cells transfected with vector plasmids and TRIM23 overexpression plasmids (n = 3, **P < .01, ***P < .001)

Gene set enrichment analysis is designed to detect co‐ordinated differences in the expression of predefined sets of functionally related genes. High‐throughput RNA‐sequencing data set of the colorectal cancer cohort from GEO and the data set from TCGA were used to perform GSEA to probe the TRIM23‐associated signal pathways in an unbiased manner. We found that TRIM23 overexpression was related to P53 and cell cycle signal pathways, and both signal pathways were associated with proliferation of cancer cells (Figure 5A,B).

### 3.5 TRIM23 regulated the proliferation of CRC cells via modulating the P53‐cell cycle signalling pathway

**FIGURE 6:**
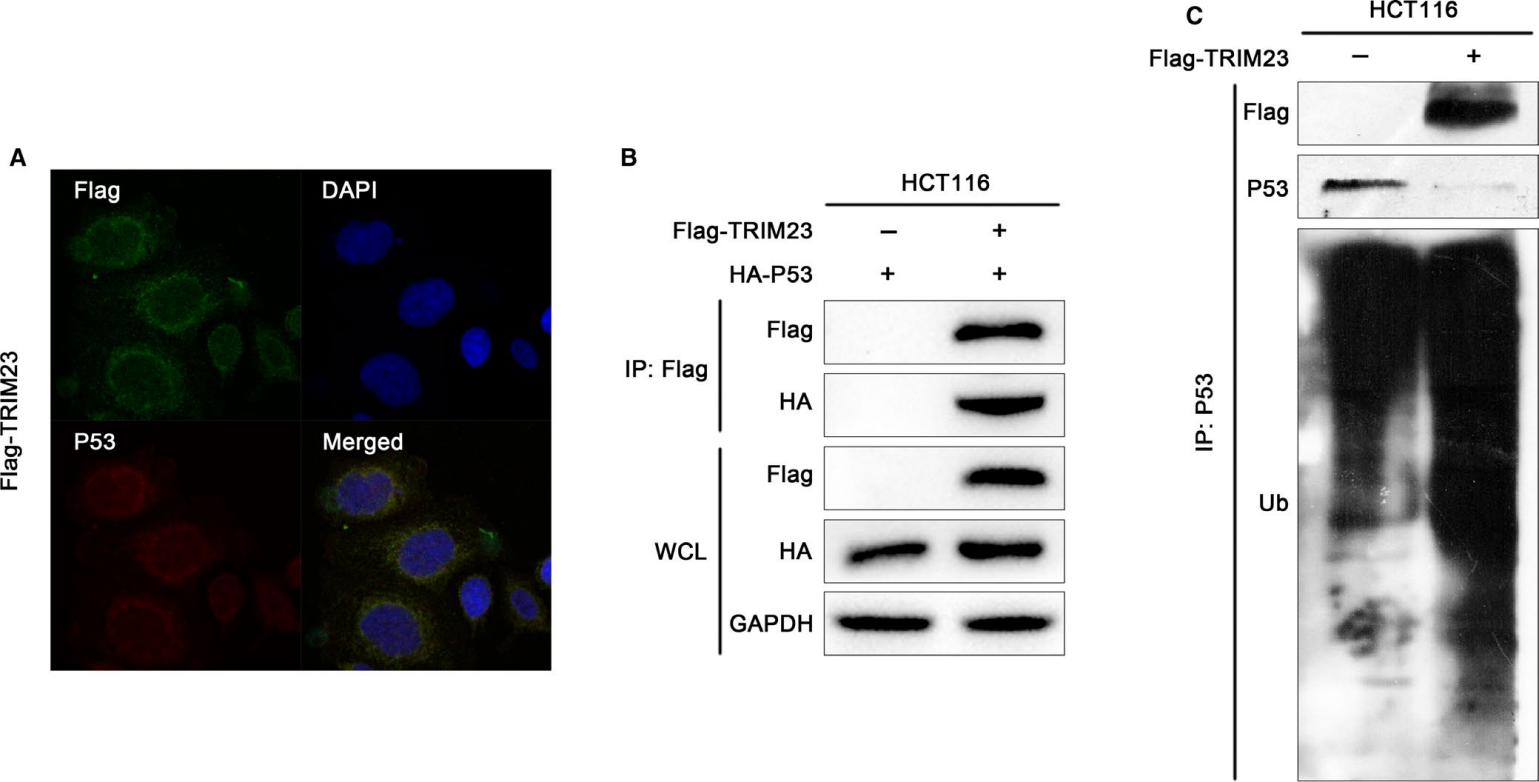
TRIM23 binds to P53, which in turn facilitates the ubiquitination and degradation of P53. A, P53 and TRIM23 co‐localize in CRC cells. B, TRIM23 co‐immunoprecipitates with P53 in HCT116 cells. C, Ubiquitination of P53 is enhanced by TRIM23 overexpression in HCT116 cells

The mRNA and protein levels of CDK4, CDK6, cyclin D1, P53 and P21, which are closely related to cell cycle and P53 signal pathways, were detected by real‐time PCR and Western blot. As shown in Figure 5C,D, the mRNA and protein levels of P21 were obviously increased, while CDK4, cyclin D1 and CDK6 levels were decreased in TRIM23‐siRNA cells compared with that in control cells. We found that the P53 protein level increased after silencing of TRIM23. Nevertheless, no change in the mRNA levels was observed in TRIM23‐siRNA cells (Figure 5C,D). In addition, we got the contrary results in HCT116 cells transfected with vector plasmids and TRIM23 overexpression plasmids (Figure 5E,F). We next investigated the molecular mechanism through which TRIM23 promoted P53 degradation. Immunofluorescent staining showed that TRIM23 and P53 co‐localized in HCT116 cells (Figure 6A). Co‐immunoprecipitation results showed that TRIM23 bound to P53 in HCT116 cells (Figure 6B). Further we observed by co‐immunoprecipitation that the ubiquitination of P53 was augmented by TRIM23 overexpression in HCT116 cells (Figure 6C). According to the results, we found that TRIM23 promoted P53 protein ubiquitination and enhanced P53 degradation. Overall, our observations indicate that TRIM23 inhibits P53 expression, accelerates cell cycle and thus enhances cell proliferation in CRC.

## 4 DISCUSSION

Accumulating studies have shown that TRIM proteins have been implicated in several cancers.^10^, ^11^, ^24^, ^25^ However, the expression pattern, functional implication and prognostic value of TRIM23 in CRC have been poorly defined. Our study provides experimental evidence that TRIM23 is overexpressed significantly in colorectal cancer, and the immunostaining intensity of TRIM23 correlates positively with lymph node metastasis, tumour size and AJCC stage (Table 1). Obviously, shortened overall survival is seen in patients with high TRIM23 expression compared with those with low TRIM23 expression. Multivariable Cox regression analyses revealed that, along with AJCC stage, the TRIM23 overexpression (*P* = .027) could be considered an independent prognostic factor for CRC (Table 2). In cultured CRC cells and xenograft nude mouse models, depletion of TRIM23 significantly suppresses CRC cell proliferation. Stated thus, TRIM23 is suggested as a potent biomarker for evaluation of the prognosis of patients with CRC.

Most TRIM proteins have ubiquitin E3 ligase activity and lead to the degradation of proteins.^26^ The exact signalling pathway that TRIM23 may regulate in colorectal cancer remains unclear. Gene set enrichment analysis showed that P53‐cell cycle signalling pathways were positively associated with TRIM23 expression. As a known tumour suppressor, P53 participates in various critical biological processes and plays crucial roles in CRC development.^27^, ^28^ The P53 expression is regulated by various E3 ubiquitin ligases,^29^ including TRIM proteins, such as TRIM24, TRIM25, TRIM29 and TRIM59.^16^, ^30^, ^31^, ^32^ As shown in our result, knock‐down of TRIM23 can induce G1‐phase arrest and inhibit cell proliferation (Figure 4). P21 is an momentous molecule downstream of P53, which negatively regulates the expression of CDK4/6 and cyclin D1 in cell cycle pathway.^33^ CDK4/6 can trigger G1 entry from quiescence and facilitate G1 progression by combining with cyclin D1 and then promoting cancer cell proliferation.^34^ We find that TRIM23 decreases the P53 protein level and regulates its downstream molecules (P21, cyclin D1 and CDK4/6); however, no change in mRNA levels of p53 was observed, suggesting a post‐transcriptional regulation of P53 by TRIM23. Considering the ubiquitin E3 ligase activity in a large number of TRIM family proteins, we found that TRIM23 physically binds to P53 and enhances the ubiquitination and degradation of P53 (Figure 6), then accelerates cell cycle and enhances cell proliferation. Nevertheless, further research is needed to explore the specific mechanism in the future.

In summary, our study has shown the clinical and biological significance of TRIM23 in colorectal cancer. TRIM23 exerts an inhibitory effect on P53 and its downstream molecules, and then promoting cell proliferation and tumour growth. TRIM23 may be regarded as a useful prognostic factor and a potential treatment target for CRC.

## CONFLICT OF INTEREST

The authors confirm that there are no conflicts of interest.

## AUTHOR CONTRIBUTIONS

Yudong Han, Haijun Lu and Haiying Tian conceived and designed the study. Yudong Han and Ye Tan performed the experiments. Yuanyuan Zhao, Yongchun Zhang and Xinjia He analysed the data. Li Yu and Haiping Jiang revised the manuscript. All authors approved the final version of the manuscript.

## Supporting information

Table S1

## Data Availability

The data used to support the findings of this study are available from the corresponding author upon request.
