# Supplementary material for: TRIM23 overexpression is a poor prognostic factor and contributes to carcinogenesis in colorectal cancer
Source: J Cell Mol Med. 2020 Mar 30;24(10):5491–500. doi: 10.1111/jcmm.15203 (PMC7214184; doi:10.1111/jcmm.15203)
Supplement: Supplementary file 1 — Table S1 [file JCMM-24-5491-s001.docx]

**Supplementary TABLE S1** Primers for real-time RT-PCR

| **Primer sequences (5****′→3′)** | |
| --- | --- |
| TRIM23 | 5′ ATGTTGCTGGAGCACTGTCA 3′ |
|  | 5′ GAGAGCCAGTCCAACCCTTC 3′ |
| P53 | 5′ AACGGTACTCCGCCACC 3′ |
|  | 5′ CGTGTCACCGTCGTGGA 3′ |
| P21 | 5′ TGGAGACTCTCAGGGTCGAAA 3′ |
|  | 5′ GGCGTTTGGAGTGGTAGAAATC 3′ |
| Cyclin D1 | 5′ TGGAGGTCTGCGAGGAACA 3′ |
|  | 5′ TTCATCTTAGAGGCCACGAACA 3′ |
| CDK4 | 5′ ACTGGCCTCGAGATGTATCC 3′ |
|  | 5′ TGCTGCAGAGCTCGAAAGGC 3′ |
| CDK6 | 5′ CGTGGTCAGGTTGTTTGATGTG 3′ |
|  | 5′ ACTCGGTGTGAATGAAGAAAGTCC 3′ |
| GAPDH | 5′CACCCACTCCTCCACCTTTG3′ |
|  | 5′CCACCACCCTGTTGCTGTAG3′ |
